# The Importance of Tau Phosphorylation for Neurodegenerative Diseases

**DOI:** 10.3389/fneur.2013.00083

**Published:** 2013-07-01

**Authors:** Wendy Noble, Diane P. Hanger, Christopher C. J. Miller, Simon Lovestone

**Affiliations:** ^1^Department of Neuroscience, King’s College London, Institute of Psychiatry, London, UK; ^2^Department of Neuroscience and Clinical Neurosciences, King’s College London, Institute of Psychiatry, London, UK; ^3^Department of Old Age Psychiatry, Institute of Psychiatry, King’s College London, London, UK

**Keywords:** tau, phosphorylation, oligomers, Alzheimer’s disease, function, extracellular

## Abstract

Fibrillar deposits of highly phosphorylated tau are a key pathological feature of several neurodegenerative tauopathies including Alzheimer’s disease (AD) and some frontotemporal dementias. Increasing evidence suggests that the presence of these end-stage neurofibrillary lesions do not cause neuronal loss, but rather that alterations to soluble tau proteins induce neurodegeneration. In particular, aberrant tau phosphorylation is acknowledged to be a key disease process, influencing tau structure, distribution, and function in neurons. Although typically described as a cytosolic protein that associates with microtubules and regulates axonal transport, several additional functions of tau have recently been demonstrated, including roles in DNA stabilization, and synaptic function. Most recently, studies examining the trans-synaptic spread of tau pathology in disease models have suggested a potential role for extracellular tau in cell signaling pathways intrinsic to neurodegeneration. Here we review the evidence showing that tau phosphorylation plays a key role in neurodegenerative tauopathies. We also comment on the tractability of altering phosphorylation-dependent tau functions for therapeutic intervention in AD and related disorders.

## Introduction

Characteristic accumulations of highly phosphorylated tau protein aggregates are found in several neurodegenerative tauopathies including Alzheimer’s disease (AD), progressive supranuclear palsy (PSP), corticobasal degeneration (CBD), and some forms of frontotemporal lobar dementia (FTLD-tau). It was assumed that these pathological tau aggregates are the toxic form of tau. However, recent studies indicate that soluble and highly phosphorylated tau species are more closely associated with synaptic dysfunction and cell loss (1–4).

**Figure 1:**
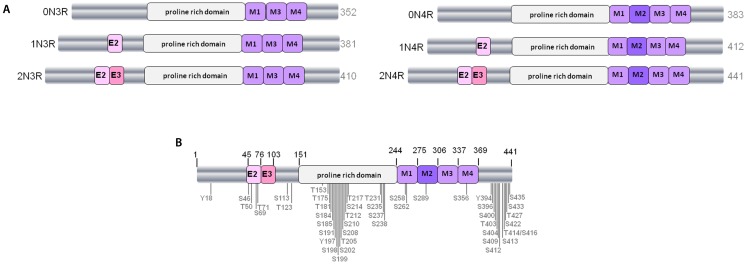
**The human tau gene and six protein isoforms**. **(A)** The six isoforms of human CNS tau. Exons 2 and 3 (E2 and E3) encode two different inserts of 28 amino acids near the N-terminus of tau. Absence of E2 and E3 gives rise to 0N tau isoforms, whereas inclusion of E2 produces 1N and inclusion of both E2 and E3 results in 2N tau isoforms. M1–M4 represent the four imperfect-repeat microtubule-binding domains, M2 being encoded by exon 10. Lack of M2 results in the formation of 3R tau and M2 inclusion results in 4R tau isoforms. The proline-rich domain in the center of the tau polypeptide is indicated. Alternative-splicing produces tau isoforms of 352–441 amino acids, as indicated. **(B)** Positioning of phosphorylation sites on tau from Alzheimer brain. Approximately 45 phosphorylation sites have been identified, these are found predominantly in the proline-rich domain and the regions flanking the microtubule-binding domain.

Tau is normally a highly soluble protein found predominantly in neurons. A total of six different isoforms of tau are expressed in the adult human CNS via alternative splicing of the MAPT gene, which comprises 16 exons and is found on chromosome 17q21.3. Regulated inclusion of exons 2 and 3 gives rise to tau isoforms with 0, 1, or 2 N-terminal inserts, whereas exclusion or inclusion of exon 10 leads to expression of tau isoforms with three (3R) or four (4R) microtubule-binding repeats (Figure 1A). In normal human brain the ratio of 4R–3R tau is approximately one, whereas in many tauopathies, this ratio is altered; PSP, corticobasal degeneration (CBD), and argyrophilic grain disease all exhibit over-expression of 4R tau isoforms, whereas Pick’s disease is mainly characterized by tau inclusions rich in 3R tau isoforms (5–9).

Tau is a phosphorylated protein, containing 85 potential serine (S), threonine (T), and tyrosine (Y) phosphorylation sites. Many of the phosphorylated residues on tau are found in the proline-rich domain of tau, flanking the microtubule-binding domain (Figure 1B). Both the phosphorylation status and isoform expression of tau are developmentally regulated and both are important factors for cytoskeletal plasticity during embryogenesis and early development. In early developmental stages a single tau isoform, 0N3R, is expressed and tau phosphorylation is elevated relative to adult brain. In contrast, all six tau isoforms are present in normal mature human brain, and at this stage tau phosphorylation is relatively reduced (8, 10).

Despite the significant heterogeneity that exists between and within the various tauopathies, the deposited tau in pathological lesions is invariably highly phosphorylated. Mass spectrometric analysis, combined with Edman sequencing and specific antibody reactivity, shows that approximately ten phosphorylation sites can be detected on soluble tau purified from normal brain (10). In contrast, when insoluble aggregated tau is extracted from tauopathy brain, at least 16 phosphorylated residues have been found in PSP (11–13), and approximately 45 different serine, threonine, and tyrosine phosphorylation sites, representing more than 50% of all phosphorylatable residues, have been found in AD brain (10, 14–17).

A large number of different kinases and phosphatases have been shown to regulate tau phosphorylation, and an imbalance in tau kinase and phosphatase activity is believed to result in tau hyperphosphorylation in disease. Tau kinases include:

- The proline-directed kinases glycogen synthase kinase-3 (GSK-3) (18–22), cyclin-dependent kinase 5 (cdk5) (23–25), and 5′ adenosine monophosphate-activated protein kinase (AMPK) (26, 27).
- Non-proline-directed kinases, such as casein kinase 1 (CK1) (10), microtubule affinity-regulating kinases (MARKs) (28–30), cyclic AMP-dependent protein kinase A (PKA) (31, 32), and dual specificity tyrosine-phosphorylation-regulated kinase 1A (DYRK-1A) (33, 34).
- Tyrosine kinases including Fyn (35, 36), Abl (37, 38), and Syk (39).

In addition, several phosphatases dephosphorylate tau, including protein phosphatase-1, -2A, and -5 (PP1, PP2A, and PP5) (reviewed by (40).

Importantly, many of these enzymes have been implicated in pathways affected by amyloid-beta (Aβ) in models of AD (27, 41–43). It remains to be established if the overall phosphorylation state of tau or phosphorylation at specific residues is important in disease pathogenesis, as suggested by studies in flies (44). However, there is evidence that phosphorylation of individual residues on tau can significantly impact its function, and this is discussed below.

## The Relationship between Phosphorylation and Tau Structure

In addition to abnormal phosphorylation, tau protein in neurodegenerative disease brain can be modified in a number of ways, including N- and C-terminal proteolytic cleavage, altered conformation, nitration, glycosylation, acetylation, glycation, ubiquitylation, *O*-GlcNAcylation, aggregation, and filament formation (45, 46). Much research has focused on elucidating the relationship between phosphorylation and the changes in tau structure that are common in neurodegenerative disease brain. Evidence from this research suggests that phosphorylation occurs either prior to, or at the same time as, these other post-translational modifications and before aggregation occurs. It remains to be seen whether this temporal precendence indicates a causative relationship.

### Proteolytic tau cleavage

Tau is subject to proteolytic cleavage by caspase-3 at aspartate (D) residue 421 (47), and N-terminal cleavage by calpain-1 (48) and caspase-6 (49). The tau fragments that are generated have been detected in affected regions of human tauopathy brain (47, 50). Caspase-cleaved tau fragments show an increased propensity to aggregate, and these may form a seeding nidus that promotes the aggregation and fibrillization of full-length tau species (51). In contrast, cleavage of tau by calpain may partially inhibit tau aggregation (50). The temporal relationship between tau cleavage and phosphorylation is unclear, with data showing that phosphorylation of different tau residues precedes (52), follows (47), and inhibits (53) the proteolytic cleavage of tau by caspase-3. However, substantial evidence shows that caspase-3-cleaved tau species are particularly prone to phosphorylation in both primary neuronal cells (54) and human tauopathy brain (47), and that phosphorylated and caspase-3-cleaved tau species readily form aggregates in cells (55). These results therefore suggest that phosphorylation and caspase-mediated cleavage of tau are important events during the development of the characteristic tau aggregates that accumulate in AD and other tauopathies.

### Altered tau conformation

Tau is a natively unfolded protein that adopts abnormal conformations in tauopathy brain. For example, tau cleavage by caspase-3 at D421 occurs early in disease development, following an alteration in tau conformation detected by the Alz50 antibody, and prior to the formation of the conformational Tau-66 epitope (tau residues 155–244 and 305–331) which is detected in late-stage AD (56). Altered tau conformation is suggested to be a major determinant in inducing tauopathy development *in vivo* (57), and abnormal tau conformers are detected in mouse models of tauopathy where elevated tau phosphorylation is apparent, but prior to the appearance of substantial tau aggregation (22, 58). Thus, caspase-3-induced tau cleavage appears to occur relatively early during the development of tauopathies, contemporaneous with increased phosphorylation and altered conformation of tau.

### The development of tau oligomers

A number of soluble and insoluble tau oligomers have been detected in AD and FTLD brain (2). Tau oligomers display altered conformation (59), are formed during the early stages of tau aggregation (59), and are closely associated with neurodegenerative phenotypes (2, 60). For example, transgenic mice that conditionally express a proline to leucine mutation at residue 301 (P301L) in human tau (1) exhibit high molecular weight tau oligomers, prior to the presence of neurofibrillary tangles (NFTs), that correlate with the development of cognitive deficits (2). Similarly, in a *Drosophila* model of tauopathy, the suppression of tau-induced neurodegeneration is associated with clearance of ubiquitinated and phosphorylated low molecular weight (<250 kDa) tau oligomers, concomitant with increases in ubiquitinated tau monomers and high molecular weight (>250 kDa) tau oligomers (61). It should be noted that protection from tau-associated toxicity in this latter study was also accompanied by reduced phosphorylation of soluble monomeric tau. Phosphorylation of tau by GSK-3 promotes the formation of insoluble oligomeric tau species that can constitute both full-length and truncated tau species (62, 63). The majority of insoluble tau in AD brain is intact (13). However, cleaved tau species are prominent in insoluble tau preparations from PSP, CBD, and FTLD-tau brain (13). The increased propensity of caspase-cleaved tau to aggregation (47), and the close association of tau fragments with cell death (64), suggests that although present as a relatively small pool of total tau, cleaved tau may also play an important role in disease. The presence of phosphorylated oligomeric tau species in cortical synapses extracted from AD brain (65) supports a role for highly phosphorylated tau multimers in tau-associated neuronal dysfunction.

### The formation of insoluble tau aggregates

In cell-free systems, soluble tau is a hydrophilic, unstructured, and dynamic protein (66). However, highly ordered aggregated tau filaments constitute the characteristic neurofibrillary lesions observed in tauopathy brain, including NFTs in AD and FTLD-tau, astrocytic plaques in CBD and tufted astrocytes in PSP (67).

There is substantial evidence that tau phosphorylation precedes its aggregation. Highly phosphorylated mouse and human tau undergoes self-assembly *in vitro* (68, 69), and dephosphorylation of soluble tau from AD brain inhibits its polymerization and restores the ability of tau to stabilize microtubules (70). Transgenic mice in which tau kinase activity is increased display increased tau phosphorylation prior to the presence of tau aggregates (24, 25, 58, 71). Furthermore, treating tau transgenic mice with kinase inhibitors results in reduced tau phosphorylation and also a reduced tau aggregate load (22, 72, 73). It should be noted, however, that reduction of tau aggregate load in tau transgenic mice following lithium treatment could result from enhanced autophagy in addition to reduced GSK-3-mediated tau phosphorylation (74). The relationship between tau phosphorylation and aggregation is clearly complex since phosphorylation of tau at specific sites, that are known to result in tau detachment from microtubules, can prevent tau aggregation (75). In addition, disruption to tau phosphatase activity in transgenic mice leads to the development of early disease-like tau abnormalities (76, 77). In particular, tau phosphorylation at the AT100 epitope is apparent in mice with reduced PP2A activity (77), which show cdk5-mediated enhanced activation of GSK3. Phosphorylation at the AT100 site has previously been shown to precede NFT formation (78), thus these findings may also suggest that changes in tau phosphorylation precede its aggregation. However, NFT formation was lacking in mice with reduced PP2A activity, an event attributed to increased clearance of abnormal tau conformers (77).

It is possible that the formation of a small pool of cleaved tau may be critically important in mediating the formation of pathological tau aggregates. Caspase-cleaved tau is prone to phosphorylation at specific epitopes (47, 54) and forms aggregation seeds that sequester full-length tau (51). Indeed, *in vivo* imaging of tau transgenic mice has demonstrated that truncated tau induces the misfolding of soluble tau and leads to the accumulation of hyperphosphorylated tau in tangles (79). Whether or not filamentous tau aggregates are toxic, protective, or inert remains an issue of intense debate (for review, see 80). However, small aggregated tau species have attracted interest recently because of their reported involvement in the propagation/transmission of tau pathology, and this topic is discussed in more detail below.

## The Influence of Phosphorylation on Tau Localization and Function

Tau is ubiquitously expressed during early embryonic development, but becomes localized predominantly in axons of mature neurons. The mechanisms underlying the axonal sorting of tau are not fully understood, but might involve selective trafficking of tau mRNA or protein into axons (81, 82), a retrograde transport barrier in the axon initial segment in mice (83), upregulation of tau mRNA translation in axons (84) or selective degradation of tau in dendrites (85). Tau is also found in association with neuronal membranes, in the nucleus, dendrites and synapses, and extracellularly. The localization of tau is altered in disease states. In particular, the redistribution of hyperphosphorylated tau to the somatodendritic compartment is considered a hallmark pathological marker during early tauopathy development (86, 87). The functional consequences of tau phosphorylation-mediated changes in the cellular localization of tau are discussed below.

### Cytoplasmic tau: Cytoskeletal integrity and axonal transport

A large proportion of tau is found in the cytosolic compartment, where it interacts with microtubules through its C-terminal microtubule-binding domain (Figure 1, residues 244–368). The binding of tau with microtubules is regulated by tau phosphorylation status, with *in vitro* phosphorylation of recombinant tau at S262 and S356, orthologous residues in adjacent microtubule-binding repeats, reducing tau interactions with microtubules and rendering tau less susceptible to aggregation (75). Phosphorylation of tau at residues outside of the microtubule-binding domain of tau, including S214 and T231, have also been shown to reduce its interaction with microtubules (75, 88). These findings suggest that phosphorylation at different tau sites may have opposing effects on the ability of tau to aggregate Furthermore, interaction of the peptidyl-prolyl isomerase Pin1 with phosphorylated T231 mediates the interaction of PP2A with the *trans* configuration of phosphorylated tau, and results in a conformational change that restores the ability of tau to bind to microtubules (89–91). Regardless of the particular sites involved, increased tau phosphorylation that causes tau to detach from microtubules leads to the disassembly of microtubules and disruption to the structure of the neuronal cytoskeleton. In addition, the accumulation of unbound hyperphosphorylated tau in the cytoplasm could cause further microtubule disassembly by sequestering normal tau and other microtubule-associated proteins (92). When tau is in a filamentous state, its interaction with normal (soluble) tau and its inhibition of microtubule stabilization is disrupted (93). Preventing microtubule instability in tauopathies has become an important target for drug development (94, 95).

Alterations in tau phosphorylation also affect its anterograde axonal transport. In general, reducing tau phosphorylation at S/T residues decreases, whereas mimicking tau phosphorylation increases, the rate of axonal tau transport in fly, rodent, and human neurons (21, 96–98). The influence of tau phosphorylation on its transport appears to be associated with differential binding of S/T phosphorylated tau to the molecular motor protein kinesin-1 (97, 98) and differential degradation rates of phospho-tau species through the lysosomal autophagy system (98).

The interaction of tau with microtubules is critically involved in the regulation of microtubule-dependent axonal transport (99), therefore tau phosphorylation also plays a key role in regulating the transport of other important cargoes. Increasing tau phosphorylation at N-terminal Y residues relieves the inhibition of anterograde axonal transport observed in the presence of highly phosphorylated tau aggregates in squid axons (100). However, tau is not usually highly phosphorylated in squid axons and therefore it is unclear whether this provides a good model to examine mammalian tau functions. In mice over-expressing FTLD-tau mutations, there is impaired anterograde axonal transport of vesicles containing the dopamine-synthesizing enzyme tyrosine hydroxylase, which precede the loss of dopaminergic neurons in the substantia nigra (101). The transport deficits reported in this mouse model were shown to be mediated by interactions between phosphorylated tau and JNK-interacting protein 1 (JIP-1) (102). Since JIP-1 regulates the binding of cargo to kinesin, these results further support the idea that increasing tau phosphorylation disrupts axonal transport. Alternatively, reduced degradation or clearance of aggregated or mutant forms of tau might contribute to a “clogging” of microtubules and consequent disruption in axonal transport (103).

Disruption to axonal transport is predicted to be an early event in several neurodegenerative diseases (104) and recent evidence suggests that dysregulated axonal transport may contribute to tau-induced degeneration. Genetic suppression of Miro, an adapter protein essential for mitochondrial axonal transport, exacerbates the neurodegenerative phenotype in *Drosophila* expressing human tau, through a mechanism dependent upon phosphorylation of tau at S262 by PAR-1, the *Drosophila* homolog of MARK kinase (105). Similarly, deletion of kinesin light chain-1 results in accumulation of hyperphosphorylated tau and the appearance of axonal spheroids in mice (106), in line with numerous reports that have characterized the binding of tau to kinesin (21, 96–98).

Finally, alterations in mitochondrial transport and function are intrinsically linked with several neurodegenerative diseases (107). Over-expression of tau *in vivo* results in alterations to mitochondrial distribution that are associated with soluble, rather than fibrillar, tau species (108). In addition, tau phosphorylation alters the axonal transport and distribution of mitochondria in cultured neuronal cells (109, 110), an effect recently attributed to tau phosphorylation-dependent changes in inter-microtubule spacing (110). Furthermore, highly phosphorylated tau has been shown to interact with the mitochondrial fission protein, Drp1 (111), and DuBoff et al. (112) demonstrated that this relationship is important for neurodegeneration. They show that actin is over-stablised in *Drosophila* that express human tau, and that this impairs the actin-based translocation of Drp1 and mitochondria, which reduces their interaction and leads to accumulation of Drp1 on F-actin, mitochondrial elongation, and downstream neurotoxicity (112). Thus tau phosphorylation is closely linked to alterations in the localization and/or function of mitochondria. It is therefore likely that phosphorylated tau influences synaptic dysfunction in tauopathies by contributing to the depletion of functional mitochondria from synapses (113).

### Membrane-associated tau: A cell signaling role for tau?

Tau interacts with several neuronal membranes, including the endoplasmic reticulum (114), the Golgi network (114), and the plasma membrane (115, 116). An increasing body of evidence shows that the association of tau with plasma membranes is regulated by phosphorylation (116–118). Plasma membrane-associated tau is dephosphorylated at several sites known to be aberrantly phosphorylated in AD brain (116, 117, 119, 120). Indeed, phosphorylation of tau at N-terminal, but not C-terminal, residues prevents its membrane localization in tau-transfected cells, demonstrating that the phosphorylation state of tau directly impacts its positioning at membranes (116).

Tau has also been detected within cell-surface lipid-rich microdomains of the plasma membrane (35, 41, 121), and the amount of tau associated with these lipid rafts is regulated by tau phosphorylation at N-terminal tyrosine residues (121). Tau interactions with the non-receptor tyrosine kinase Fyn are critical for the interaction of tau with lipid rafts (35, 41, 121) and neuronal plasma membranes (116). Tau can interact with Fyn via its SH2 and SH3 domains (121, 122). Phosphorylation of tau at Y18 is important for tau interactions with Fyn-SH2 (121), whereas phosphorylation of S/T residues on tau negatively influences its interaction with Fyn-SH3 (122). Accumulating evidence therefore suggests that targeting of tau to the plasma membrane may be regulated by the interaction of the tau N-terminal projection domain with the SH3 or SH2 domains of tyrosine kinases such as Fyn (118). Furthermore, these data suggest that by binding to several important signaling molecules in a manner that is regulated by phosphorylation, tau has the potential for a broad role in cell signaling (122).

### Dendritic tau and synaptic toxicity

A number of recent cell and animal studies have shown an important role for tau in dendrites leading to the suggestion that tau-mediated synaptic dysfunction may be one of the earliest events in the pathogenesis of tauopathies. Several studies have indicated that the presence of tau aggregates is detrimental to synaptic health (123, 124), however, soluble tau species are associated with synapse loss in mouse models of tauopathy (125) and phosphorylated tau oligomers have also been detected in synapses in postmortem AD brain (126).

A small amount of tau exisits in dendrites under normal conditions, where it acts to target Fyn post-synaptically, regulating *N*-methyl-d-aspartate (NMDA) receptor subunit 2 phosphorylation and interactions between NMDA receptors and the post-synaptic density protein PSD-95 (3). Disease insults, such as increased concentrations of Aβ in AD, lead to the detachment of highly phosphorylated tau from microtubules and its accumulation in intact dendritic spines (3). This in turn causes local elevations in Ca^2+^ and disruption of synaptic function through impaired trafficking and/or synaptic anchoring of glutamate receptors (3, 127, 128). In a related study, the redistribution of hyperphosphorylated tau into dendritic spines led to reductions in α-amino-3-hydroxy-5-methyl-4-isoxazolepropionic acid (AMPA) receptor subtypes that caused impairments in basal synaptic transmission and long term potentiation (129). Thus, there is increasing evidence that tau-mediated synaptic dysfunction might be one of the earliest events in the pathogenesis of tauopathies (reviewed by 130). Therefore, correction of aberrant tau phosphorylation may be therapeutically beneficial during very early stages of disease progression when synaptic deficits first develop. In this respect, it is worth noting that inhibition of GSK3 has previously been shown to attenuate deficits in LTP (131).

### Nuclear tau – a role in DNA protection

It was first suggested that tau might have novel functions mediated by interactions with DNA or RNA following observations that tau is present in the nuclei of human neuroblastoma cells (132). Full-length tau was identified in neuronal nuclei, where it colocalizes with the chromosome scaffold, nuclear and nucleolar organization centers and can exist as SDS-insoluble species (132, 133). Further studies revealed that the microtubule-binding domain of tau can bind RNA (134), and single and double stranded DNA (135, 136). The interaction of tau with RNA enhances tau polymerization; the RNA acting as a nucleation center for tau aggregation (134), whereas interaction of tau with DNA results in conformational changes in DNA (133) and suppression of DNA amplification *in vitro* (136). Insights into the nuclear function of tau were recently revealed with the observation that tau protects DNA from heat damage and oxidative stress (137). Nuclear tau appears to be largely dephosphorylated (137), suggesting that increased tau phosphorylation in diseased states could interfere with protective functions of non-phosphorylated tau in neuronal nuclei.

### Extracellular tau and the propagation of tau pathology

Tau is present in brain insterstitial fluid in the absence of any neurodegeneration (138). Recent evidence suggests that this extracellular tau is likely to have important functional consequences for neuronal health and for the spread of tau pathology across the brain during disease progression.

To allow investigation of tau pathology spread *in vivo*, transgenic mice have been created with neuropsin-promoter targeted expression of tau in layer II neurons of the entorhinal cortex. These mice demonstrate an age-dependent spread of phosphorylated and aggregated abnormal tau confomers from the site of transgene expression to neighboring neurons and anatomically connected brain regions (139, 140). There are several mechanisms that could account for this observed spread of tau pathology. Firstly, degenerating neurons with high levels of transgene expression might release pathological forms of tau that subsequently propagate in a “prion-like” fashion through their uptake by neighboring neurons. In support of this process, Frost et al. (141) demonstrated that extracellular tau aggregates, but not tau monomers, are taken up by cultured human embryonic kidney (HEK293) cells and neuronal stem cells, leading to fibrillization of full-length intracellular tau. Similarly, small oligomers of tau, similar to those found in human tauopathy brain, can be taken up by cultured neuronal cells via bulk endocytosis (142). It is possible that this process also underlies the postulated prion-like transmission of tau pathology to distal brain regions observed when pathological forms of human tau are injected into mice expressing wild-type human tau (143, 144). Secondly, tau pathology in the neuropsin-promoter regulated tau transgenic mice appears to spread to anatomically connected pathways in the absence of any notable cell loss (139, 140), suggesting that tau is released from intact neurons and then taken up by connected cells. This process is supported in part by recent findings showing endogenous tau release from cultured neurons in the absence of cell death (145, 146). Interestingly, the release of endogenous full-length tau from rat primary neurons was shown to be a dynamic and physiological process that is calcium-dependent and stimulated by AMPA receptor activation and neuronal activity (146), suggesting that tau release may play a role in signaling between neurons. Indeed, exogenously applied tau can interact with muscarinic receptors on the surface of cultured neuronal cells, promoting increases in intracellular calcium that alter cell signaling pathways (147). It is also possible that tau propagation may be mediated via glial cells, since cytosolic tau accumulations are observed in neurons surrounded by activated microglia (148) and astrocytes promote tau phosphorylation in neighboring neurons (54).

The relationship between tau secretion and tau phosphorylation state is not yet established. However, extracellular tau released from primary neurons, neuroblastoma cells and non-neuronal cells is dephosphorylated at several epitopes known to be highly phosphorylated in AD brain (145, 146, 149) and this has been proposed to result from the action of extracellular tissue non-specific alkaline phosphatase (149). How this relates to the phosphorylation state of intracellular tau is not clear, although the secretion of C-terminally cleaved tau from non-neuronal cells can be enhanced by the increased phosphorylation or cleavage of intracellular tau (150). These studies indicate that changes in tau phosphorylation can modulate its release from neurons, and therefore is also likely to influence the effects of extracellular tau on neuronal health and the spread of tau pathology in diseased brain.

## Tau Phosphorylation as a Therapeutic Target

**Figure 2:**
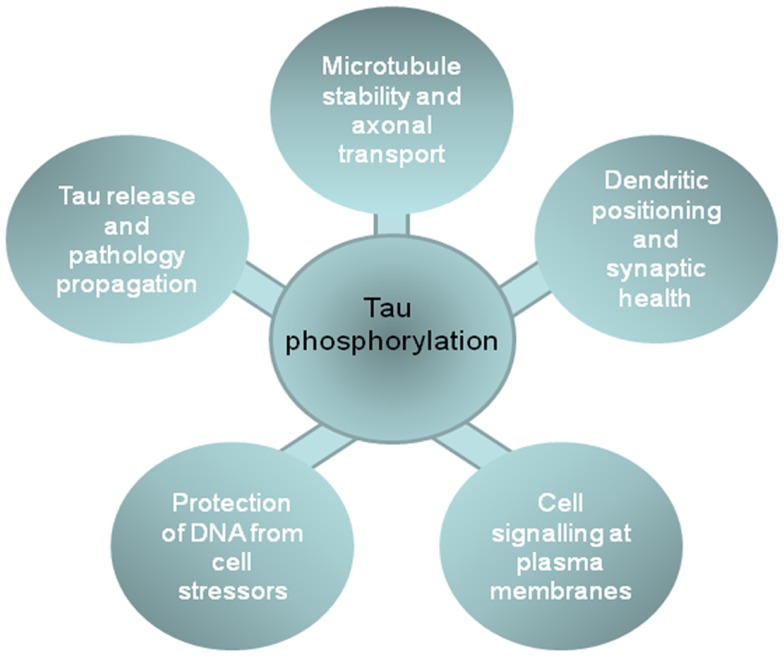
**The impact of phosphorylation on tau functions in different cell locations**. The figure shows the functions of tau in different cellular compartments that are influenced by tau phosphorylation, and that likely contribute to the development or progression of neurodegenerative tauopathies.

As summarized above, tau phosphorylation plays a key role in regulating tau function at different neuronal locations, including the involvement of cytosolic tau in stabilizing the neuronal cytoskeleton and influencing axonal transport; the role of membrane tau and extracellular tau in cell signaling and neurofibrillary pathology spread through diseased brains; the relationship between nuclear tau and protection from DNA damage; and dendritic functions of tau that are involved in synaptotoxicity (Figure 2). These data suggest that inhibition of tau phosphorylation could have widespread disease-modifying effects in tauopathies. Therapeutic strategies aimed at targeting tau phosphorylation have been widely reviewed elsewhere (e.g., 8, 9, 67, 151), therefore we will comment only briefly here.

Although several kinases and phosphatases regulate tau phosphorylation, only GSK-3 inhibitors have entered clinical trials for the treatment of AD or rarer tauopathies such as PSP. Based on promising data from animal models (21, 22, 152), the relatively non-specific GSK-3 inhibitor, lithium, was tested in small-scale clinical trials for mild to moderate AD. Whilst lithium did not cause significant adverse effects in an open label study of a year (153), neither did it have any beneficial effects in a short-term trial (154). However, a small trial of lithium in patients with mild cognitive impairment reduced phosphorylated tau in CSF and reported better performance of treated patients in cognitive and attention tasks (155), suggesting that administration of lithium during the early stages of disease could have some therapeutic benefit in defined patient populations.

Tideglusib (NP-12) is a non-ATP competitive inhibitor of GSK3 that has entered clinical trials. Tideglusib has disease-modifying effects when administered to transgenic mice that develop both tau and amyloid pathology (156). Pilot trials for tideglusib in AD and PSP showed good tolerance of tideglusib (157) and phase II studies are underway.

Kinase inhibitors have entered clinical use for conditions unrelated to neurodegeneration (158). However, kinases make for complex therapeutic targets, and probably because of incomplete drug specificity, off-target effects are problematical. An alternative strategy may be to modulate the activity of proteins that directly affect the activity of tau kinases. One interesting target in this respect is lemur tyrosine kinase-2 (LMTK2). LMTK2 phosphorylates PP1C on T320, thereby inhibiting PP1C activity (159–161). PP1 regulates phosphorylation of GSK3β at the inhibitory phosphorylation site S9 (162, 163), and therefore, via its effect on PP1C, LMTK2 regulates GSK-3β phosphorylation at S9, and ultimately GSK-3 activity (160, 161). Therefore, an alternative strategy for inhibiting GSK-3 activity may be to increase LMTK2 expression or activity. Small molecule allosteric agonists for a variety of kinases have now been described, and the development of kinase agonists has been identified as key area for the development of new therapies (164).

Finally, biomarkers are increasingly used to follow the progression of AD, and in some cases to support early diagnosis of the disease (165). However, to accelerate the clinical translation of therapeutics that modify tau phosphorylation, it is essential that sensitive and specific biomarkers are available to allow the measurement of drug–target interactions, and the impact of treatment on downstream pathophysiology. The development of such target validation biomarkers will allow a faster selection of candidate treatments, and appropriate dose ranges. This should accelerate the clinical development of tau phosphorylation inhibitors that are likely to have wide-ranging benefit for the treatment of AD and related tauopathies.

## Conflict of Interest Statement

The authors declare that the research was conducted in the absence of any commercial or financial relationships that could be construed as a potential conflict of interest.
